# The Epidemiology of Chronic Suppurative Lung Disease and Bronchiectasis in Children and Adolescents

**DOI:** 10.3389/fped.2017.00027

**Published:** 2017-02-20

**Authors:** Gabrielle B. McCallum, Michael J. Binks

**Affiliations:** ^1^Child Health Division, Menzies School of Health Research, Charles Darwin University, Darwin, NT, Australia

**Keywords:** chronic suppurative lung disease, bronchiectasis, children, epidemiology, etiology

## Abstract

In the modern era, the global burden of childhood chronic suppurative lung disease (CSLD) remains poorly captured by the literature. What is clear, however, is that CSLD is essentially a disease of poverty. Disadvantaged children from indigenous and low- and middle-income populations had a substantially higher burden of CSLD, generally infectious in etiology and of a more severe nature, than children in high-income countries. A universal issue was the delay in diagnosis and the inconsistent reporting of clinical features. Importantly, infection-related CSLD is largely preventable. A considerable research and clinical effort is needed to identify modifiable risk factors and socioeconomic determinants of CSLD and provide robust evidence to guide optimal prevention and management strategies. The purpose of this review was to update the international literature on the epidemiology, etiology, and clinical features of pediatric CSLD.

## Introduction

Bronchiectasis, a chronic progressive disease of the airways, remains one of the most neglected diseases in respiratory health (1). It is characterized by abnormal dilatation of the bronchi caused by protracted inflammation (2) and by chronic productive or wet cough (3). A definitive diagnosis of bronchiectasis requires a chest high resolution computer tomography (cHRCT) (4), with cases otherwise referred to as having chronic suppurative lung disease (CSLD) (5). Bronchiectasis or CSLD (from hereafter combined and referred to CSLD) has multiple etiologies and are often associated with underlying conditions (e.g., congenital malformation, cystic fibrosis, or immune deficiency) (6, 7). However, recurrent acute lower respiratory infections (ALRI) during early childhood, a crucial time for lung growth and development, are arguably the common etiology for CSLD, particularly among socially disadvantaged children (8, 9).

Globally, the prevalence of CSLD in high-income countries over the last 50 years has declined with the introduction of antibiotics, immunizations, improved hygiene, nutrition, and access to medical care (10–16). However, a substantial burden of CSLD persists among socially disadvantaged populations of high-income countries (e.g., Alaskan, Australian, Canadian, Maori, and Pacific Islander children) (8, 16–20) with the extent of pediatric CSLD in low- and middle-income countries largely unknown. In recent years, there has been a growing awareness of CSLD related to the increased use of cHRCT diagnostics and emerging research into the etiology (6), microbiology, immunology (21), and clinical management (22), yet, robust epidemiological data remain sparse. Our understanding of the complex interplay between the host, pathogens, and the environment is largely superficial, and there have been few clinical intervention trials among children and adolescents. Here, we provide an update on the epidemiology, etiology, and clinical features of pediatric CSLD not associated with cystic fibrosis.

## Global Incidence of CSLD

### Historical

French physician Rene Laennec first described CSLD in the early nineteenth century (23), with the first surgery (24) and imaging (25) performed around 100 years later. In the 1930s, Roles described the poor prognosis of CSLD patients at the time and highlighted the importance of early diagnosis and the potential of lobectomy for enhanced survival (26). The benefit of surgery over medical management was unclear in several subsequent studies (27–30), though a surgical case series of CSLD conducted decades later was convincing, particularly for children (13). With the introduction of broad spectrum antibiotics in the 1950s, significant reductions in CSLD incidence were reported. In the United Kingdom (UK) between 1952 and 1960 (12), CSLD admissions fell fivefold from 23.8 to 4.9 per 1,000 total admissions. Similar findings were reported elsewhere in the UK (10) and in the United States of America (USA) (11). Childhood CSLD became confined to disadvantaged populations of high-income countries, earning it the label of an “orphan disease” (14). Alaskan native children <16 years of age appeared vulnerable with a conservatively estimated case prevalence of 41/100,000 population over the decade 1956–1966, largely in the Yukon–Kuskokwim (YK) Delta and with one-third of cases associated with tuberculosis infection (16). In the 1960s, the prevalence of CSLD among Scottish children (<10 years of age) was 10.6/100,000 (27), and in the early 1970s, a substantial burden of CSLD was reported among indigenous children living in Central Australia (31). In this study, 83 indigenous children with CSLD were identified (60% <2 years of age) from an estimated population of around 1,000, despite near complete eradication of tuberculosis, measles, and pertussis, previously considered to be key etiological pathogens (31). In one of the few African studies, 70/1,150 patients consecutively admitted to the University Hospital in Nigeria with respiratory or cardiovascular disease between 1975 and 1979 were diagnosed with CSLD, of which 10 (14%) were children (32).

In the following section, we review the population incidence of pediatric CSLD in the modern era (1980–2016), when immunization and antibiotic interventions control many of the major etiological pathogens of the past, including tuberculosis, pertussis, measles, and pneumococcus.

### Modern Era: 1980–2016

We performed a systematic literature review of the PubMed library followed by a bibliography search within all relevant articles. The PubMed search terms: (CSLD OR bronchiectasis) AND (pediatric OR child OR children OR infant) AND (epidemiology) yielded 351 articles, which was reduced to 243 when confined to non-cystic fibrosis-related bronchiectasis articles published since 1990 and performed after 1980, human research, and non-review articles written in English. From these, 13 relevant studies reported CSLD rates: either directly (incidence rate) or indirectly (included a bronchiectasis disease numerator, an estimate of the population denominator, and an observation period). In one New Zealand (NZ) study, age-specific data could not be accurately extracted or obtained from the authors and were, therefore, excluded (33). A government report capturing similar national NZ CSLD data was included in its place (34).

In our defined modern era (1980–2016), the burden of childhood CSLD remains difficult to characterize. In the 1980–1990s, wheezing and persistent cough were often considered clinical features of asthma and not related to CSLD. Thus, due to lack of robust evidence, misclassification was likely and management (e.g., antibiotics) possibly suboptimal. Peer reviewed published burden data were available from only nine countries, diagnostic criteria varied, population denominators were often approximate, and incidence/prevalence data were inconsistently reported. Further, hospital-based studies utilizing International Classification of Diseases coding to identify CSLD (ICD8 518, ICD9 J494, and ICD10 J47) were common. ICD coding does not provide radiological information (though several studies confirmed cHRCT diagnosis *via* medical records or subsequently performed cHRCT) nor distinguish between primary and repeat admissions (exacerbations), which must be ascertained by diagnosis in conjunction with date of presentation. While rare in most high-income countries, in some settings (e.g., Australian indigenous children) as many as 1 in 136 children have a new case of CSLD detected annually (8). Despite limitations in data accuracy, the trends toward higher rates of CSLD among socially disadvantaged populations were clear though perhaps partially attributed to enhanced surveillance. The collated global epidemiology data are summarized in Table 1.

**Table 1:** **Burden of CSLD in children**.

Reference	Pub. year	Country	Region	Population	Era	Time (years)	Male: female	Age (years)	Data source	Given or extrapolated^h^ BE cases (*n*)	Chest high resolution computer tomography (*n*)	Median age at diagnosis (years)	Given or rate extrapolated^b^ population denominator (*n*)	Alternative^b^ population denominator estimate (*n*)	Given or extrapolated^i^ average annual incidence
**Affluent countries**															
Saynajakangas et al. (35)	1998	Finland	National	Non-specific	1983–1992	10	31:16	<14	Hospital admissions (ICD8 518; ICD9 494)	47	na	na	**959,184^a^**	944,253^c^ (1983–1992avg)	0.5

Dawson and Bakalinova (36)	1997	UAE	Al Ain	Arabic	1994–1995	1	na	1–13	Pediatric hospital clinic	12	na	na	**90,000**	nr	13.3^i^

Laverty et al. (37)	2008	UK	All countries	Non-specific	2006–2007	1	na	<16	Electronic registry	23	na	na	na	**11,644,416^c^** (2006^)^	0.20^i^

Zaid et al. (38)	2010	Republic of Ireland	National	Non-specific	2006	1	na	<18^g^	Pediatrician surveillance	24^h^	24	na	na	**1,040,623^c^** (2006)	2.3

Simpson et al. (34)	2014	NZ	National	Non-specific	2009–2013	5	na	<15	Hospital admissions (ICD10 J47)	681	na	na	**908,000**	1,000,160^c^ (2013)	15.0

**Disadvantaged populations**															
Flynn (19)	1994	Fiji	Suva	Native Fijian	1985–1989	4	na	5–14	Hospital admissions (ICD9 494)	25	na	na	**89,285^a^**	78,960^d^ (1994)	7.0^j^

Singleton et al. (16)	2000	USA	Alaska (YK Delta)	Alaskan natives	1980–1990	10	na	<14^g^	Statewide registry and hospitalizations	~91^h^	28^+^	na	na	6,500^e^ (1990)	~140^j^

Edwards (18)	2003	NZ	Auckland	TOTAL Pacific Island Maori Europeans Other	1998–2000	3	36:24	1–17	Hospital admissions	60 33 15 8 4	60	8.0	**354,000^a^** 60,180 63,720 173,460 56,640	307,600^f^ 57,000 50,600 167,000 33,000 (2001)	5.7^j^ 18.3 7.9 1.5 2.4

Chang et al. (8)	2003	Australia	Central	Indigenous	2000–2002	2	31:34	≤15	Hospital admissions (ICD10 J47) + medical record review	65	59	5.4	**4,422^a^**	nr	735.0^i^

Twiss et al. (20)	2005	NZ	National	TOTAL Pacific Island Maori European Other	2001–2002	2	28:37	≤15	Pediatrician surveillance	63 32 19 18 3	63	5.2	**851,351^a^** *89,887* *197,916* *600,000* *62,500*	877,200^f^ *100,000* *216,100* *652,600* *69,000* (2001)	3.7 17.8 4.8 1.5 2.4

O’Grady et al. (39)	2010	Australia	NT	Indigenous	1999–2004	5	7:3	<1	Hospital admissions (ICD10 J47)	10	na	0.7	**9,295**	nr	118

Das and Kovesi (17)	2014	Canada	Qikiqtani, Nunavut	Indigenous	1998–2011	13	na	<17	Medical record review	17	17	5.6	**8,415^a^**	nr	15.5^i^

Janu et al. (40)	2014	Australia	Central Qld	Indigenous	2007–2011	5	4:3	<2	Hospital admissions (ICD10 J47) + medical record review	7	7	0.5	**341^a^**	nr	410

*Incidence standardized to an annual average per 100,000 children. Repeat bronchiectasis episodes were excluded from hospital data where possible in an attempt to focus on the index cases. Most studies reported a study observation period rather than actual person–time of observation censored at the bronchiectasis event. Censoring of these rare events in large populations would have had little effect on incidence. Given population denominators were included where possible, otherwise ^a^rate extrapolated population denominators were calculated (from numerator and rate)*.

*^b^Alternative population denominators were sourced where necessary and to reaffirm rate estimated population denominators*.

*^c^World Population Prospects: the 2015 Revision. United Nations, Department of Economic and Social Affairs, Population Division. http://populationpyramid.net (accessed 2/11/2016)*.

*^d^Fiji census data, 1986. http://www.statsfiji.gov.fj/statistics (accessed 2/11/2016)*.

*^e^Status of Alaskan Natives Report (41) and Alaska Native Health Status Report (42)*.

*^f^Statistics New Zealand. http://nzdotstat.stats.govt.nz (accessed 7/11/2016)*.

*^g^Estimated age range based on a smaller bronchiectasis study sub-population than that which the rate calculation was based on*.

*^h^Extrapolated numerator calculated from the given or estimated denominator and the given rate*.

*^i^Extrapolated average annual incidence calculated from the numerator, given or estimated denominator, and the study observation period*.

*^j^Extrapolated average annual incidence rate in this case was per 100,000 children born between 1980 and 1990 and followed until 1998*.

*na, not available; nr, not relevant*.

*~, data estimated from a graph and unable to be confirmed by the authors*.

*Bold indicates the population denominator matched to the incidence calculations*.

*Italics highlight sub-category data that are not mutually exclusive (i.e., sums to greater than the total)*.

### High-Income Countries

In most affluent European populations, pediatric CSLD was relatively uncommon with average annual rates ranging from 0.2/100,000 in the UK (37) to 2.3/100,000 in Ireland (38), however, in the equally affluent, yet non-European United Arab Emirates (UAE; 13.3/100,000) (36) and largely non-European NZ (15.0/100,000) (34), CSLD was more common. The indigenous Arabic children in the UAE children were predominantly from a wealthy social demographic with almost universal immunization coverage, therefore, the surprisingly high rates may be related to an inherited predisposition or to moderate levels of educational attainment. In NZ, the high national rate is a reflection of the high proportion of less affluent Maori and Pacific Islander people (~40%) for which there is a well-established susceptibility to respiratory infection (19, 43, 44). In this particular report by Simpson (34), CSLD cases were not reported by ethnicity. As clarified in the next section, the annual rate was much lower (1.5/100,000) for NZ people of European heritage (18, 20).

### Socially Disadvantaged Populations of High-Income Countries

The highest reported pediatric CSLD rates occur among socially disadvantaged indigenous populations of the Pacific Islands (19), NZ (18, 20), Australia (8, 39, 40), Alaska (16), and Canada (17). In the Pacific, Native Fijian children (5–14 years of age) had over 20-fold more CSLD hospital admissions (7.0 versus ~0.3/100,000) than their Indo-Fijian counterparts between 1985 and 1989 (19). Two NZ studies spanning 1998–2002, one examining national CSLD cases reported by pediatricians (20) and the other CSLD hospital admissions in Auckland (43), demonstrated the disproportionate average annual incidence among Maori (4.8–7.9/100,000) and Pacific Islander (17.8–18.3/100,000) compared to European children (1.5/100,000). This disparity is directly related to child poverty. In NZ, substantially more Maori and Pacific Islander families live in poor (28–34%), overcrowded (25–50%) households with high unemployment rates (15%) compared to European families (16, 5, and 5%, respectively) (34). Similarly, in Australia and Alaska, CSLD is largely confined to indigenous children.

In the early 2000s, the average annual incidence of cHRCT confirmed bronchiectasis among indigenous children living in Central Australia was high at 735/100,000 (8). Almost all children (95%) had pneumonia at an early age (median 6 months) and nearly two-thirds had concurrent chronic suppurative otitis media (8). Two further hospital-based studies have investigated CSLD among Australian indigenous children. The first study, a historical cohort examining the entire indigenous population of Australia’s Northern Territory (NT) (both Central and Northern Australia from 1999 to 2004) showed an average CSLD incidence of 118/100,000 child-years in the first 12 months of life (39). One in five of these infants were also hospitalized with an ALRI. The early burden of respiratory illness is of particular concern in this region. The second study, a retrospective chart review conducted during 2007–2011 at the Mount Isa Base Hospital in Queensland (Qld), demonstrated an average annual incidence of 410/100,000 in children <2 years of age (40).

Alaskan Natives are also historically at high risk of CSLD. In a conglomeration of CSLD data from the YK Delta region (collated from a state-wide registry, historical and current patients of YK Delta Regional Hospital, or Alaska Native Medical Center), the average annual incidence of CSLD among children born between 1980 and 1990 (the review defined modern era) was 140/100,000 (16); equivalent to the incidence reported among the same population born in the 1940s (16). For a similar population, the Inuit children of Nunavut, Canada, the average annual incidence (1998–2011) of pediatric cHRCT confirmed bronchiectasis ascertained *via* health record reviews was lower but also substantial at 15.5/100,000 (17).

Nationally in NZ (20), and among indigenous children of Australia (8), Alaska (16), and Canada (17), CSLD was diagnosed at around 5 years of age. In Auckland though, the age was slightly older at 8 years (18) and under 2 years of age for Australian indigenous infants (39, 40). Common to disadvantaged populations of high-income countries, was low socioeconomic status, inadequate and overcrowded housing, complex environmental and social issues, limited access to health care, frequent exposure to camp or cooking fires, high smoking rates, and almost a universal history of early infant pneumonia and/or recurrent ALRIs (8, 16–20, 39, 40). In affluent countries, CSLD is more frequently associated with non-infectious etiology (see “Etiology”).

Published pediatric CSLD data (etiological, management, diagnostic, etc.) are widespread. Countries including Turkey (45–48), Saudi Arabia (49), Taiwan (50), Malaysia (51), Tunisia (52), Italy (53, 54), and England (55) have published excellent research (etiological, management, diagnostic, etc.), however, population-incidence could not be accurately elucidated or was not the focus of these research articles. We did not identify any incidence data among low- to middle-income countries.

## CSLD-Related Mortality

There are limited data on CSLD mortality in the pediatric population. More deaths occur in adults. In Central Australia, a hospital record review showed that 34.2% (41/120) of indigenous adults diagnosed with CSLD 2001–2007 died during that same period. Most had a history of pneumonia, nearly half were diagnosed in childhood and the median age of death was 42.5 years (56). A study in England and Wales showed that only 0.2% (12/5,745) of total CSLD deaths recorded between 2001 and 2007 occurred among children aged <14 years (57). Whereas, a NZ audit of an electronic CSLD database (1991–2006), found that 7% (6/91) of children diagnosed with CSLD (median age 7.3 years) died during follow-up (median follow-up 6.3 years) (58). A more recent study in India identified 80 children (mean age 9.6 years) with CSLD *via* medical record reviews. Among 62 children who were prospectively followed for 1 year, 5 (11%) died. To avoid premature death from CSLD in both children and adults, it is vital to intervene as early as possible.

## Etiology and Clinical Manifestations of Pediatric CSLD

A systematic literature review of the PubMed library using the search terms: (CSLD OR bronchiectasis) AND (pediatric OR child OR children OR infant) and restricted by time (post 1990), type (non-review), language (English only), and availability, yielded 1,163 articles. Further screening to exclude cystic-fibrosis, HIV related, surgical, small (<10 CSLD cases), overlapping, or biased CSLD populations refined the list to 28 articles (Table 2). From these publications, 26 contributed etiology (Table 3) and 27 clinical manifestation data (Table 4).

**Table 2:** **Demographics of CSLD from pediatric studies**.

Reference	Country	Region	Era	*N*	M:F	Age of onset of first respiratory symptoms in years, median (range)	Age at diagnosis of bronchiectasis in years, median (range)
**High-income countries**							
Nikolaizik and Warner (59)	UK	London	1994	41	na	na	na
Li et al. (60)	UK	London	1986–2002	136	65:71	na	na
Kapur et al. (61)	Australia	Qld	1992–2009	113	64:49	na	5.3 (range 2.7–7.9)
Eastham et al. (55)	UK	Newcastle	1996–2002	93	62:31	1.1 (0–16)	7.2 (1.6–18.8)
Zaid et al. (38)	Ireland	Dublin	1996–2006	92	42/50	3.9 (1–12)	6.4 (1.5–13)
Santamaria et al. (54)	Italy	Naples	2001–05	105	50:55	0.5 (0.08–8.5)	7 (0–14.4)

**Socially disadvantaged populations of high-income countries**							
Singleton et al. (16)	Alaska	Alaska (YK delta)	1998	46	na	0.4 (0–4.8)	4.8 (1–15)
Edwards et al. (18)	NZ	Auckland	1998–2000	60	36:24	1 (0–14)	8 (na)
Chang et al. (8)	Australia	Central	2000–02	59	29:30	0.5 (0–10)	5.4 (0.7–15)
Twiss et al. (20)	NZ	National	2001–02	65	28:37	2.3 (0–14)	5.2 (0.5–15)
Singleton et al. (9)	Australia	NT, SA, Qld	2004–10	97	55:39	0.31 (0–3.9)	na
	USA	Alaska (YK delta)	2004–10	41	22:19	0.2 (0–0.8)	na
	NZ	Auckland	2008–10	42	25:17	0.5 (0.1–4.2)	na
Munro et al. (58)	NZ	National	2011	91	49:50	na	7.3 (0.9–16)
Das and Kovesi (17)	Canada	Qikiqtani, Nunavut	2015	17	na	na	5.7 (1.6–15.6)

**Low- and middle-income countries**							
Karadag et al. (47)	Turkey	na	1987–2001	111	56:55	2.5 ± 2.7^b^	7.4 ± 3.7^b^
Karakoc et al. (45)	Turkey	Southern	1993–99	23	13:10	na	6.2 ± 3.6^b^
Lai et al. (50)	Taiwan	Northern	1991–2001	29	12:17	na	na
Bouyahia et al. (52)^a^	Tunisia	Tunis	1994–2006	41	na	~3.1 (na)^b^	5.8 (0.5–14)
Banjar (49)	Saudi Arabia	Riyadh	1993–2005	151	75:76	2.3 ± 2.2^b^	7.3 ± 4.1^b^
Koh et al. (62)	Korea	Seoul	1995–96	25	14/11	na	na
Kim et al. (63)	Korea	Seoul	1999–2008	92	47/45	na	7.6 (0.2–18)
Dogru et al. (46)	Turkey	Ankara	na	204	105:99	2.3 ± 2.2^b^	8 (na)
Babayigit et al. (48)	Turkey	Izmar	2003–08	66	44/22	na	na
Nathan et al. (51)	Malaysia	Kuala Lumpur	2004–12	60	43/17	0.5 (0–8)	1.3 (0.2–11)
Kumar et al. (64)	India	New Dehli	2006–13	80	50/30	na	9.6 (2–15)
Gokdemir et al. (65)	Turkey	Istanbul	2011–12	47	21/22	3.4 ± 3.3	na
Bahali et al. (66)	Turkey	Istanbul	2013	76	32/44	5.1 ± 4.6	na

*Adapted from Kapur et al. (67) and Brower et al. (6) and additional studies found (as able)*.

*^a^Unable to access full article*.

*^b^mean ± SD*.

*na, not available or not described*.

*~, estimated from provided data*.

**Table 3:** **Etiology of CSLD from pediatric studies**.

	Postinfection (%)	Immune deficiency (%)	Primary ciliary dyskinesia (%)	Congenital malformations (%)	Aspiration (%)	Idiopathic (%)	Other (%)
**High-income countries**							
Nikolaizik and Warner (59)	32	27	17	15	5	2	2
Li et al. (60)	4	34	15	4	18	26	0
Eastham et al. (55)^b^	35	26	1	9	5	18	14
Zaid et al. (38)	17	22	9	1	22	32	3
Kapur et al. (61)	12	12	2	na	11	55	8
Santamaria et al. (54)	7	10	24	na	4	55	0

**Socially disadvantaged populations of high-income countries**							
Singleton et al. (16)	93	na	na	na	4	na	na
Edwards et al. (18)	25	12	0	na	10	50	3
Chang et al. (8)	90	3	0	1	5	0	2
Twiss et al. (20)	22	6	0	0	6	54	11
Munro et al. (58)	23	9	na	na	na	45	23
Das and Kovesi (17)	94	0	0	na	6	na	12

**Low- and middle-income countries**							
Karadag et al. (47)	30	15	6	3	4	38	4
Karakoc et al. (45)	35	17	13	na	na	na	34^c^
Lai et al. (50)	28	10	3	3	7	31	18
Bouyahia et al. (52)^a^	10	10	10	na	na	48	22^c^
Banjar (49)	na	18	11	7	10	40	14
Koh et al. (62)	24	na	24	na	na	52	0
Kim et al. (63)	21	9	4	na	na	14	65^d^
Dogru et al. (46)	16	5	12	na	3	49	15
Babayigit et al. (48)	21	8	6	3	9	33	17
Nathan et al. (51)	40	7	na	10	na	18	na
Kumar et al. (64)	24	6	15	4	3	36	na
Gokdemir et al. (65)	19	19	26	na	2	33	na
Bahali et al. (66)	16	4	20	na	na	53	8

*Adapted from Kapur et al. (67) and Brower et al. (6)*.

*^a^Unable to access full article*.

*na, not available or not described*.

*Etiology is described where available*.

*^b^Multiple etiologies occurred in some children, therefore, the sum is >100%*.

^c^Includes cystic fibrosis

*^d^Bronchiolitis obliterans (33%) and interstitial lung disease (17%) were common*.

**Table 4:** **Clinical features reported in children with CSLD**.

	Cough (%)	Wheeze (%)	Chest deformity (%)	Clubbing (%)	Hemoptysis (%)	Failure to thrive (FTT) (%)	FEV1% predicted, median (range)	FVC% predicted, median (range)	Chest pain (%)	Dyspnea (%)
**High-income countries**										
Nikolaizik and Warner (59)	na	na	na	na	na	na	na	na	na	na
Li et al. (60)	35	10	na	na	na	4	71 (15–133)	77 (14–22)	na	na
Kapur et al. (61)	na	na	na	na	na	na	na	na	na	na
Eastham et al. (55)	na	na	na	na	na	na	na	na	na	na
Zaid et al. (38)	na	na	na	na	na	na	na	na	na	na
Santamaria et al. (54)	na	na	na	na	na	na	95 (26–144)	96 (30–132)	na	na

**Socially disadvantaged populations of high-income countries**										
Singleton et al. (16)	na	na	na	na	na	17	na	na	na	na
Edwards et al. (18)	na	na	60	52	na	8	69 (36–110)^b^	86 (33–109)^b^	na	na
Chang et al. (8)	100^c^	na	60	26	na	73	66.2 (38–98)	70.2 (40.2–110)	na	na
Twiss et al. (20)	40	na	na	na	na	na	77 (na)	85 (na)	na	na
Singleton et al. (9)	50	7	14	10	na	82	na	na	na	30
	75	41	18	5	na	95	na	na	na	58
	52	17	57	45	na	74	na	na	na	26
Munro et al. (58)	na	na	42	41	na	15	66 (18–116)^b^	72 (17–123)^b^	na	na
Das et al. (17)	59	na	na	na	na	12	78 (63–108)	na	na	na

**Low- and middle-income countries**										
Karadag et al. (47)	97	47	15	41	10	na	63.3 (22.1)^b^	67.3 (23.1)^b^	na	50
Karakoc et al. (45)	91	48	na	na	na	na	68.45 (13.70)^b^	70.34 (9.56)^b^	na	57
Lai et al. (50)	93	35	na	21	41	na	67.6 (43.8)^b^	82.5 (39.1)^b^	na	10
Bouyahia et al. (52)^a^	na	na	27	27	5	na	na	na	na	34
Banjar et al. (49)	>66	>66	na	33	5	>66	na	na	na	na
Koh et al. (62)	28	28	na	na	na	na	83 (7)^b^	na	na	28
Kim et al. (63)	50	20	na	4	8	na	63 (na)	71 (na)	3	25
Dogru et al. (46)	83	na	1	13	4	46	na	na	na	9
Babayigit et al. (48)	100	20	5	23	5	27	na	na	na	na
Nathan et al. (51)	na	na	na	na	na	na	52 (32–76)	58 (37–76)	na	na
Kumar et al. (64)	96	53	na	na	16	10	na	Na	43	na
Gokdemir et al. (65)	na	na	na	na	na	na	79.8 (20.6)^b^	80.0 (17.8)^b^	na	na
Bahali et al. (66)	na	na	na	na	na	na	72.0 (21.9)^b^	76.4 (20.0)^b^	na	na

*Adapted from Kapur et al. (67)*.

*^a^Unable to access full article. Signs and symptoms overlap often totaling >100%*.

*^b^Mean ± SD and/or range*.

na, not available or not described; FEV_1_, forced expiratory volume in first second; FVC, forced vital capacity

*^c^Study inclusion specifically >4 months daily moist and/or productive cough. FTT was inconsistently defined. In the Singleton study (9), FTT was reported as ≤2 SD below norm*.

### Demographics

The 26 studies of pediatric CSLD encompassed 2,103 children from 15 countries around the world; 13 high-income (including studies within disadvantaged sub-populations) and 13 low- and middle-income countries (Table 2). The age of symptom onset varied significantly among the studies (range 0–16 years) but was consistently present many years prior to a diagnosis of CSLD [Table 2; mean/median differences ranging from 0.8 to 7 years (Table 2)]. Some of the earliest reported symptoms were among indigenous infants from Australia (0.3years) (8), and Alaska (0.2 years) (9), and New Zealand (0.5 years) emphasizing the early origins of CSLD in high-risk children. Interestingly, Malaysian (low and middle income) and Italian (high income) children had a similar age of symptom onset (0.5 years). Thus, early symptom identification is essential to preventing CSLD progressing and long-term decline in lung function (68–70).

As with studies reporting incidence (several of which overlap), the demographic, etiological, and clinical data varied in their nature relating to differences from study design, study era (e.g., clinical reporting) and the age at diagnosis of children involved (e.g., unable to perform lung function <4 years).

### Etiology

Chronic suppurative lung disease is the end result of chronic airway inflammation that is driven by persistent infection (2). As such, the natural history of CSLD depends largely on the susceptibility to both acute and chronic infection (71). Historically, congenital malformations, cystic fibrosis, immune deficiency, and aspiration are common antecedents (5), although a primary cause is not always identified. Further, measles, tuberculosis, and pertussis (27, 72), once the most common causes of CSLD may be less important today in the wake of advances (5) in vaccinations, antibiotics, and access to health care (67). Reviews of CSLD etiology from pediatric cohorts have been published in 2011 (67) and 2014 (6). Using a systematic search, we further updated the etiology literature, which remains similar as previous reviews and is briefly summarized below (Table 3) (17, 38, 48, 51, 58, 61, 63–66).

Less than half of the CSLD reported in high-income countries (4–35%) (38, 54, 55, 59–61, 73) and low- and middle-income countries (10–40%) (45–52, 62, 63) was identified as postinfectious; yet, a history of early infant pneumonia and/or recurrent ALRIs was almost universally common to indigenous children of Alaska (93%) (16), Australia (90%) (8), and Canada (94%) (17). A separate case-control study in Central Australia also found a strong relationship between severe recurrent pneumonia in early childhood and development of CSLD (74). In the South Korean study by Kim et al., 89% (17/19) children with infectious etiology had tuberculosis (63). Taken together, these findings reinforce the notion that the high burden of CSLD among indigenous children is related to substandard living conditions and is essentially preventable.

In affluent European populations where CSLD is less common, immune deficiency was identified in a larger proportion of cases (10–34%) (38, 54, 55, 59–61, 73) than among indigenous populations (0–12%) (8, 16–18, 20, 58) and in low- and middle-income countries (4–19%) (45–52, 62–66), where acute respiratory infections are endemic and CSLD is more common. Other main differences highlighted in Table 3 were the higher proportion of children with primary ciliary dyskinesia (PCD) in high- and low- and middle-income countries (1–24 and 3–26%, respectively) (38, 45–52, 54, 55, 59–63, 73) compared to indigenous children where PCD was not reported (8, 16–18, 20, 58). Despite improved diagnostics and case management globally, the proportion of CSLD cases with unknown etiology was relatively consistent (0–55%; Table 3) apart from in London (2%) (59) and among indigenous children (0%) where most cases were accounted for (8, 16). It is likely that previous infections play more of a role in the development of CSLD than is currently known. This is of particular concern in low- and middle-income countries where children with recurrent respiratory infections often have limited access to medical care. Even more concerning is that the actual burden of CSLD in these countries is largely unknown.

## Clinical Features and Outcomes

In this update of the literature surrounding clinical features of CSLD, importantly, we have included the largest prospective international multicenter study for CSLD in children (9, 75). The most comprehensive clinical data in this section are recorded among indigenous populations and low- and middle-income countries (summarized in Table 4).

Clinical features of CSLD differ depending on the extent of the disease. In CSLD, recurrent wet or productive cough is probably the earliest and most important symptom to recognize and investigate, although it should be noted that young children do not readily expectorate (3). Despite the importance of cough in respiratory health, this was only specifically reported in 59% (16/27) of studies, though we acknowledge in many studies, it may have been assumed or captured in other scoring algorithms. Low- and middle-income countries consisting of Turkey [7% (47), 91% (45), and 100% (48)], India [96% (64)], and Taiwan [93% (50)] and the indigenous cohorts of Alaska [75% (9)], Australia [100% (8)], and NZ [52% (9)] reported high rates of cough. Under-appreciating the importance of cough may delay diagnosis and treatment, which can lead to a decline in lung function and poorer long-term prognosis (76). Likewise, cough is an important clinical feature of CSLD exacerbations (1).

In low- to middle-income countries, the proportion of CSLD children presenting with wheeze ranged from 20% in Tunisia (48) and South Korea (63) to 66% (49) in Saudi Arabia, though it was unclear whether all 66% of the Saudi children had wheeze as it co-reported with other symptoms. The only high-income country reporting wheeze was the UK where 10% of children were identified. Interestingly, in the multicenter study of indigenous children (9), Alaskan natives had a much higher percentage of wheezing (41%) than either indigenous children of Australia (7%) or Pacific Islander and Maori children of NZ (17%). This correlated with an earlier study that showed both Alaskan native children with (65%) and without (37%) CSLD had higher rates of wheeze (77). It is likely that there are different underlying susceptibilities to and clinical manifestations of CSLD across population groups, but further exploration is needed to further understand these relationships.

Inadequate nutrition (macro and micro) is another factor that can increase the risk of developing and/or worsening CSLD by compromising innate and adaptive immunity (78), which can have long-term consequences (67). As an example, sufficient vitamin D is known to be important for regulating immune responses to respiratory pathogens (79). Among indigenous Australian children, deficiency is associated with an increased risk of hospitalization for respiratory infection (80). A typical feature of suboptimal energy and protein intake is failure to thrive (FTT), which was reported just over one-third of studies. Not surprisingly, FTT was most frequent in socially disadvantaged populations; indigenous children from Australia (73–82%) (8, 9), Alaska (95%), NZ (74%) (9), and low- and middle-income countries of Turkey (46%) (46) and Saudi Arabia (66%) (49).

Currently, cHRCT (the gold standard) and pulmonary function measurements are the most frequently used objective tools used to assess CSLD. Abnormal lung function over time predicts poor quality of life score (81–83). The percent predicted forced expiratory volume in 1 second (FEV_1_) reported across studies varied, but was lower among children from indigenous (8, 18) and low- and middle-income countries (8, 18, 47, 62) than for children from high-income countries (54–61, 63–72) suggesting more severely affected lung function (Table 4).

Since the previous review by Kapur et al. (67), a further two studies (17, 63) reporting lobar involvement (cHRCT) were identified, making 15 studies in total (8, 16–18, 20, 38, 45–50, 54, 55, 63). Multi-lobar involvement was reported in all 15 studies and was most common in Taiwan (50), NZ (18), Canada (17), and in Saudi Arabia (49). The number of affected lobes varied between studies. The left lower lobe (80%) and right lower lobe (60%) were most commonly affected. Whether multi-lobar involvement relates to clinical severity and poorer outcomes remains uncertain.

In more advanced CSLD, clinical features include chest wall deformity, digital clubbing, hemoptysis, and dyspnea. Again, these markers were common to indigenous populations and low- and middle-income countries (Table 4). A further feature, chest pain, was reported in two studies from Seoul (3%) (63) and India (43%) (64). It is possible that chest pain relates to a delayed diagnosis of CSLD (median age 7.6 years in Korea and 9.6 years in India) again emphasizing the need for earlier symptom surveillance and diagnosis to improve clinical outcomes for children with CSLD. In the longer term, complications associated with CSLD extend beyond the respiratory system including cardiovascular disease an area requiring further investigation (84).

While there are limited long-term clinical data, these studies indicate that children with CSLD have different clinical manifestations across populations. These data also highlights that children from socially disadvantaged groups (e.g., indigenous populations and low- and middle-income countries) appear to have more extensive clinical features and greater propensity for poorer long-term outcomes than those from high-income countries.

## Prevention and Management

Despite the high global burden of respiratory diseases, only a small percentage of research and development funding and resources are devoted to these conditions (71, 85). To prevent CSLD, a multidisciplinary approach is required beginning in the antennal period continuing throughout childhood. Key strategies include immunizations, improved hygiene, nutrition, and education (that is culturally appropriate) to improve health-related outcomes for children with CSLD (9, 86). Other fundamental factors to consider are exposure to tobacco, camp fire, biomass fuels, and other environmental air pollutants, overcrowding, housing quality, and access to running water. Evidence-based clinical guidelines have been developed in several settings to guide primary and secondary health care (22, 78, 87, 88), yet, more robust and consistent evidence from populations across the globe is essential. Other papers in this series will focus on the management of CSLD in more detail.

## Future Research

There is a need for more epidemiological research globally to clarify the prevalence of CSLD, in particular, for at-risk populations (e.g., indigenous and low- and middle- income countries). Importantly, there are also no long-term prospective studies in children to guide clinical care and management into adolescence and adulthood. Clinical trials across multiple countries are essential to further improve interventional and clinical management of CSLD. This can only be achieved with substantial investment and support from governments and funding bodies.

## Conclusion

Once considered an “orphans disease” among high-income populations, this review reinforces that CSLD remains a disease of poverty, common among children from indigenous populations and low- and middle-income countries. Delayed diagnosis was common as was severe disease. Despite the fact that the burden of CSLD in these settings was primarily related to early preventable infections, this condition is neglected globally in terms of research priority and funding. To improve the respiratory health of disadvantaged children, a concerted international effort is needed to determine and understand the burden of pediatric CSLD and to provide a solid evidence base for future clinical care and management.

## Author Contributions

All authors listed have made substantial, direct, and intellectual contribution to the work and approved it for publication.

## Conflict of Interest Statement

The authors declare that the research was conducted in the absence of any commercial or financial relationships that could be construed as a potential conflict of interest.

## Abbreviations

ALRI, acute lower respiratory infection, cHRCT, chest high resolution computed tomography scan, CSLD, chronic suppurative lung disease, FTT, failure to thrive, NT, Northern Territory, NZ, New Zealand, Qld, Queensland, PCD, primary ciliary dyskinesia, UAE, United Arab Emirates, UK, United Kingdom, USA, United States of America, YK, Yukon–Kuskokwim.
